# Pomegranate Supplementation Improves Affective and Motor Behavior in Mice after Radiation Exposure

**DOI:** 10.1155/2013/940830

**Published:** 2013-04-15

**Authors:** Melissa S. Dulcich, Richard E. Hartman

**Affiliations:** Behavioral Neuroscience Laboratory, Department of Psychology, School of Behavioral Health, Loma Linda University, Suite 106, 11130 Anderson Street, Loma Linda, CA 92350, USA

## Abstract

Currently, NASA has plans for extended space travel, and previous research indicates that space radiation can have negative effects on cognitive skills as well as physical and mental health. With long-term space travel, astronauts will be exposed to greater radiation levels. Research shows that an antioxidant-enriched diet may offer some protection against the cellular effects of radiation and may provide significant neuroprotection from the effects of radiation-induced cognitive and behavioral skill deficits. Ninety-six C57BL/6 mice (48 pomegranate fed and 48 control) were irradiated with proton radiation (2 Gy), and two-month postradiation behaviors were assessed using a battery of behavioral tests to measure cognitive and motor functions. Proton irradiation was associated with depression-like behaviors in the tail suspension test, but this effect was ameliorated by the pomegranate diet. Males, in general, displayed worse coordination and balance than females on the rotarod task, and the pomegranate diet ameliorated this effect. Overall, it appears that proton irradiation, which may be encountered in space, may induce a different pattern of behavioral deficits in males than females and that a pomegranate diet may confer protection against some of those effects.

## 1. Introduction

Astronauts have traveled outside Earth's atmosphere into lower Earth orbit and beyond for decades. However, these individuals are constantly exposed to ionizing radiation that may have deleterious effects on motor and cognitive abilities, as well as physical and mental health [1, 2] via increased oxidative stress [3, 4]. With plans to extend space travel beyond lower Earth orbit for longer durations (i.e., manned expeditions to Mars), radiation exposure will increase. A diet rich with polyphenols (which have antioxidant and other biologically beneficial properties) may confer enough protection to maintain the complex cognitive and fine motor skills required by astronauts.

Phytochemicals, including the phenols, terpenes, and organosulfurs, are nonnutritive, bioactive compounds commonly found in plants and animals and are known for their protective properties. Phytochemicals not only protect the plants that produce them against metabolic and environmental disease but also, when consumed, can provide numerous health benefits to humans [5, 6]. Foods containing phytochemicals have been shown to ameliorate cognitive and behavioral deficits resulting from various diseases [7, 8]. These findings have often been attributed to the antioxidant, antiapoptotic, and anti-inflammatory properties of phytochemicals [9]. Some fruits, for example, contain polyphenols that can impart extensive protective properties against damage from oxidative stress, neuroinflammation, and cognitive/behavioral deficits induced by radiation and/or aging [10–12]. 

Pomegranates (*Punica granatum*) have a high polyphenol concentration, and the juice may have 2-3 times greater antioxidant capacity compared to other antioxidant sources such as red wine or green tea [13–22]. Pomegranate polyphenols include flavonoids (i.e., quercetin) and tannins (i.e., punicalagins and ellagitannins). Flavonoids and tannins have specifically been shown to decrease oxidative stress by deactivating reactive oxygen species [19] and scavenging free radicals [19, 22]. Although these isolated pomegranate polyphenols have antioxidant properties when acting individually, research suggests that the whole range of polyphenols found in pomegranates combines synergistically to prevent oxidative stress-induced damage. Indeed, one study analyzing the antiapoptotic properties of pomegranate juice found the whole juice to be more effective than any one isolated component [18]. Furthermore, pomegranate juice has been found to ameliorate both amyloid-*β* plaque load as well as associated spatial learning deficits in a mouse model for Alzheimer's disease [23].

Altogether, this evidence suggests that the polyphenols found in pomegranates could provide an effective therapy against radiation-induced neurological and cognitive/behavioral deficits. No studies to date have looked at the radioprotective effects of pomegranate antioxidants; however, the evidence indicates that antioxidants may be beneficial in ameliorating the negative effects of ionizing radiation. Therefore, this study explores the putative protective effects of pomegranate juice on radiation-induced cognitive and behavioral deficits. 

## 2. Methods

Male and female C57BL/6 mice (4-month old) were obtained from Charles River laboratory and housed approximately 3–6 per cage. All mice were maintained on a 12-hour light/dark schedule and fed standard rodent chow *ad libitum*. All animals were treated in accordance with the requirements of the National Institutes of Health Guide for the Care and Use of Laboratory Animals, as well as the specifications stipulated by the Loma Linda University Institutional Animal Care and Use Committee.

Male and female mice were separated into the following groups: pomegranate/no pomegranate, radiation/no radiation (yielding a total of 8 groups). Forty-eight animals were started on a diluted pomegranate juice diet and continued on this diet throughout the experiment. Pomegranate juice (PomWonderful) was purchased from local grocery stores and kept refrigerated at 4°C throughout the experiment. The juice was diluted 1 : 20 with filtered water and administered through the animal's water bottles. The amount of polyphenols that each mouse consumed per day was estimated to be ~0.6 mg, which is roughly equivalent on a mg/kg basis to a human drinking two 8-ounce glasses of PJ per day. Forty-eight animals received sugar water that mimicked the sugar content of the diluted PJ.

After three weeks, the mice were subjected to irradiation or sham treatment. Half of the mice were placed into well-ventilated acrylic boxes and irradiated with a whole body radiation beam of proton radiation (150 MeV/n) at a dose of 2 Gy for 5 minutes at the Loma Linda University Protons Treatment Facility. The sham treatment animals were placed in the boxes for an amount of time equivalent to that of the radiation group, but no radiation was administered. The mice were not anesthetized, but movement was limited to comfortable breathing during proton irradiation. Eight weeks after irradiation, the mice were subjected to a battery of behavioral tests designed to assess a wide variety of cognitive and motor functions over a 2-week time span.

### 2.1. Water Maze Testing for Spatial Learning and Memory

The water maze is the standard test of learning and memory abilities in rodents [23, 24]. This test of spatial navigation requires the animal to learn the location of a hidden (submerged) platform in a pool of water using visual cues from around the room. The water maze consisted of a metal pool (110 cm diameter) filled with water made opaque with white tempera paint. The pool contained a round platform (11 cm diameter) that the animal could step on to escape the water. For each trial, an animal was released nose against the wall into the pool at one of four release points and allowed to swim to the platform. The animals were given 10 trials per day. All trials lasted a maximum of 60 s. When an animal did not find the platform in the allotted 60 s, the experimenter manually guided the mouse to the platform. An overhead camera recorded the animals' swim paths by a computerized tracking system (Noldus Ethovision), allowing for quantification of distance, latency, proximity to target, and swimming speed. Each animal received 10 trials on four consecutive days, making a total of 40 trials per animal. Trials were performed in blocks of 5 each day (2 trials per block), with approximately 30 min intervals between blocks. As performance improves, escape latency and swim path length generally decrease.

### 2.2. Cued Water Maze

This is a control task for assessing sensorimotor and/or motivational deficits that could affect performance during the spatial phase. For this phase, the surface of the escape platform was visible (1 cm above the surface of the water), and a pole was placed on top of the platform to make its location even more obvious. The platform's location varied from trial to trial. The animals were released into the pool opposite to the location of the platform and were allowed to remain on the platform for 5 s.

### 2.3. Spatial Water Maze

For this task, the surface of the escape platform was submerged 1 cm below the surface of the opaque water, so the mouse had to find the platform based on its relationship to the spatial cues rather than direct visualization. The location of the platform changed each day for 3 days. After finding the platform, the animals were allowed to remain on it for 5 s. At the beginning of the next day, the animals were given a “probe” trial in which the platform was removed from the water maze, and the animal was allowed to search the pool for 60 s. The amount of time spent searching the probe quadrant was measured, as well as the total number of times that the animal crossed over the former location of the platform. An hour later, the platform was placed back into the pool in its new location, and the next set of 10 trials was administered.

### 2.4. Elevated Zero Maze Testing for Anxiety-Like Behaviors

The elevated zero maze consisted of a plastic ring, 100 cm outer diameter, 10 cm wide, with 35 cm walls enclosing 2 opposing quadrants and elevated off the floor. The room lights were dimmed and halogen lights directly illuminated the open spaces of the maze. Animals were initially placed in the center of one of the open spaces, and their activity was monitored for the duration of five minutes. The percent amount of time spent within an enclosed space was calculated. Each animal received 2 trials, with the 2nd trial being conducted 48 hours after the 1st.

### 2.5. Open Field Testing for General Activity Levels/Movement Patterns

In the open field test, animals were placed in a 49 cm × 36 cm opaque open-topped plastic bin and allowed to explore for the duration of thirty minutes while the movements of each animal were recorded by an overhead camera and analyzed by a computerized tracking system (Noldus Ethovision). Various parameters were analyzed, including the distance the animal moved and the percent time spent moving. Each animal received 2 trials, with the 2nd trial being conducted 48 hours after the 1st.

### 2.6. Tail Suspension Testing for Depression-Like Behaviors

In the tail suspension test, mice were suspended by the tail with adhesive tape that was attached approximately 1 cm from the tip of the tail. The other end of the tape was wrapped around a hook embedded in the center of the ceiling of a wooden box measuring 19 cm × 21 cm and 40 cm in height. When suspended, the animal's rostral end was approximately 20 cm from the floor of the device. The box was enclosed on all sides except one (for viewing), and the room lighting and sound were kept to a minimum in order to diminish visual and acoustical disturbances. In addition, the nature of the box kept each animal visually isolated. While the mouse tried to escape its position, two assistants blinded to treatment group individually rated the mouse on immobility and agitation for the duration of 6 minutes. The time that the animal remained immobile during the final 4 minutes of a 6-minute duration was recorded [25]. Each animal received one 6-minute trial. Immobility was operationally defined as a complete lack of movement on the part of the mouse [26]. The animal was counted as immobile even if it was still swinging back and forth from a previous struggle but was now completely still in its voluntary movements. The animal was also rated immobile if it was curled up, appearing to rest while holding its front paws to its back paws, but was not currently struggling or moving. 

### 2.7. Rotarod Testing for Sensorimotor Coordination/Balance

The accelerating rotarod (Columbus Instruments) is a test of sensorimotor coordination and balance. It consisted of a 3 cm diameter rotating horizontal cylinder. The mouse was placed onto the cylinder and had to continuously walk forward to avoid falling. Latency to fall off was the dependent variable. Performance over days of testing was a measure of motor learning. The mice were tested every other day for 3 days. Three blocks of 2 consecutive trials were administered per day: 2 stationary trials (at 5 RPM steady), 2 trials that started at 5 RPM and accelerated by 3 RPM every 5 s, and 2 trials that started at 5 RPM and increased by 3 RPM every 3 seconds. Each steady trial lasted up to 60 s, and each accelerating trial lasted up to 120 s. There was approximately from 45 minutes to an hour between trials.

### 2.8. Statistical Analyses

All statistical analyses were carried out using SPSS. A mixed design ANOVA with 3 between-subjects variables (gender, pomegranate, and radiation) and 1 repeated measure (test day) was conducted on behavioral data. To control for sphericity and compound symmetry due to repeated measures in the test day (10 trials), the Huynh-Feldt correction to the degrees of freedom was used to determine the *P* value. When appropriate, univariate ANOVAs were used. An *α* level of 0.05 was used for all tests of statistical significance. An *a priori* power analysis was conducted using G*Power 3.1 to determine the number of animals necessary per group to reach a minimum power of 80% [27].

## 3. Results

**Figure 1:**
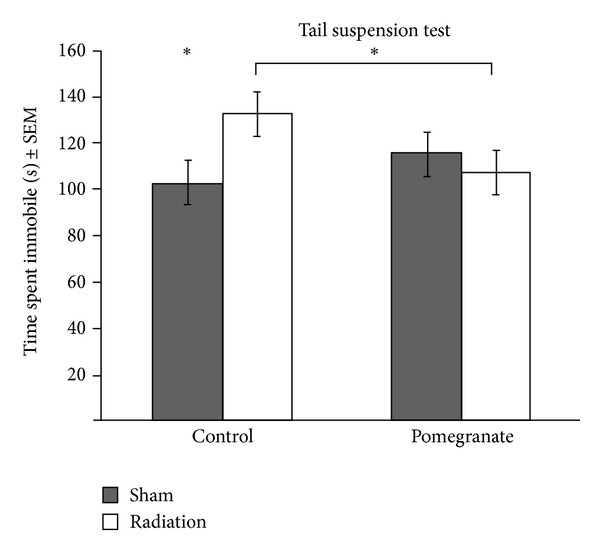
Proton radiation was associated with increased depression-like behaviors and pomegranate juice ameliorated that effect; *P < .03.

There were no effects of radiation or pomegranate treatment for the water maze (cued and spatial learning). The most striking interaction between radiation and pomegranate treatment on behavior was observed in the tail suspension test (Figure 1), which provides an assessment of depression-like behaviors. Irradiated control-fed mice exhibited more depression-like behaviors (i.e., the mice gave up sooner) than nonirradiated control-fed mice, but radiation did not have this effect on pomegranate-fed mice (*F*(1,82) = 4.92, *P* < .03). There was no difference in the performance of males versus females on this test.

**Figure 2:**
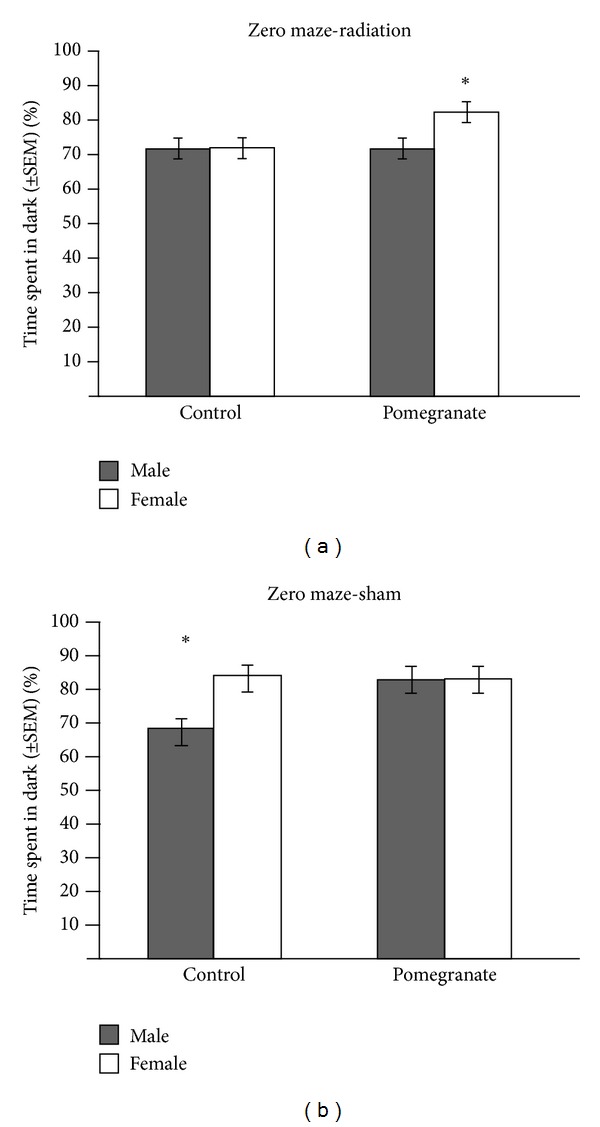
Control females spent more time in the dark than control males (suggesting heightened anxiety). (a) Radiation induced more exploration of the open quadrants in females (suggesting reduced anxiety and/or abnormal exploratory behavior), but this effect was blocked by pomegranate. (b) Interestingly, radiation did not affect males' performance, but pomegranate consumption in nonirradiated males induced behavior similar to those of females; *P < 0.004.

Radiation and pomegranate treatment had gender-specific effects in the elevated zero maze test (Figure 2), which provides an assessment of anxiety-like behaviors through exploration of an anxiety-provoking environment (*F*(1,82) = 9.19, *P* < .004). Overall, pomegranate-treated mice spent more time in the dark quadrants compared to control-treated mice (*F*(1,82) = 7.12, *P* < 0.01). Normal (nonirradiated control fed) females spent more time hiding in the dark quadrants than normal males (*F*(1,82) = 7.67, *P* < 0.008), suggesting heightened anxiety in females as compared to males. Radiation induced more exploration of the open quadrants in females, suggestive of abnormally low anxiety and/or an abnormal increase in exploratory behavior. This effect of radiation in females was blocked by the pomegranate treatment. However, both radiation and pomegranate treatment induced more time spent hiding in the dark quadrants for male mice, and thus more female-like behavior. The effect of pomegranate on these behaviors in males was attenuated by radiation exposure.

**Figure 3:**
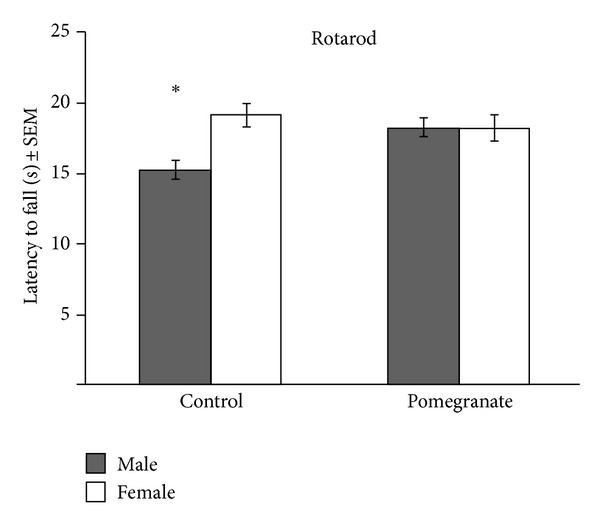
Radiation did not affect rotarod performance. Males fell off the rotating beam faster than females, but pomegranate treatment improved their performance to that of females; *P < 05.

**Figure 4:**
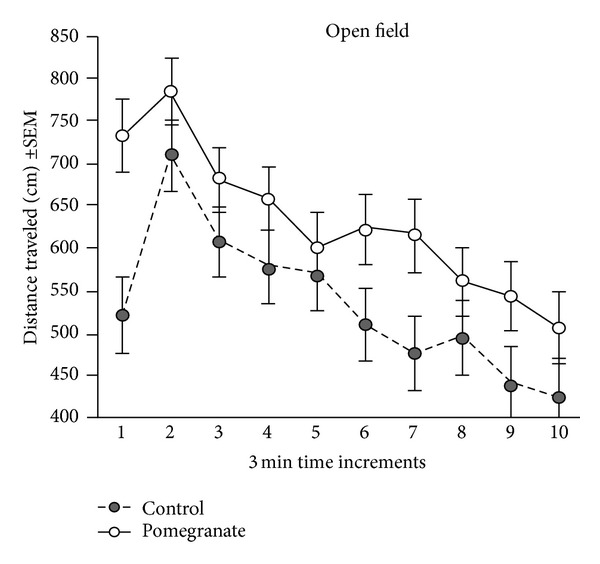
Radiation had no effect on the open field test. Pomegranate treatment increased the total distance traveled for male mice, but not female mice (data shown only for males); *P < 0.03.

Radiation had no effect on rotarod performance, which provides an assessment of sensorimotor coordination and balance. In general, males performed more poorly (fell off more quickly) than females (*F*(1,82) = 4.15, *P* < .05). Pomegranate treatment improved the performance of males to that of females (Figure 3), although it did not further improve the performance of females (*F*(1,82) = 4.20, *P* < .05). Finally, radiation had no effect on the open field test, which provides an assessment of overall activity levels and patterns. Pomegranate treatment, however, increased the total distance traveled for male mice, but not female mice (*F*(8.75,708.94) = 2.23, *P* < 0.03; Figure 4).

## 4. Discussion

The purpose of this study was to explore the putative protective effects of pomegranate juice on radiation-induced behavioral deficits in both male and female mice. Indeed, our findings demonstrated that mice exposed to radiation displayed more depression-like behaviors than nonirradiated mice but that pomegranate juice blocked this deficit. Tests that measure the latency for an animal to stop struggling in an inescapable situation are often used to measure depression-like behaviors in animal models [26]. Other studies have also reported the amelioration of depression-like behavior in animals receiving pomegranate treatment. Pomegranate extract successfully reduced depression-like behaviors in mice demonstrating ovariectomy-induced depression-like symptoms [28]. In addition, pomegranate seed extract was found to decrease depression-like behaviors similar to imipramine (an antidepressant drug) treatment in young and old mice [29].

Depression is associated with increased oxidative stress and decreased antioxidant enzyme activity, leading to subsequent DNA damage [30]. Furthermore, depression may occur in response to neuroinflammation, increased oxidative stress, decreased neurogenesis, and/or increased apoptosis [4]. Specifically, radiation-induced suppression of hippocampal neurogenesis in rodents has led to depression-like behavior [31], suggesting that the incidence of depression may increase with radiation exposure and its associated oxidative stress induction. Thus, the antioxidant and anti-inflammatory properties of pomegranate may have blocked this effect of radiation on depression-like behaviors. 

This study also demonstrated gender specific differences in anxiety-like behaviors and some interesting interactions of radiation and pomegranate juice. Control females demonstrated greater anxiety-like behaviors compared to control males on the zero maze test, spending more time in the dark enclosed quadrants. Other studies have also shown that C57BL/6 female mice spend more time in the dark enclosed arms of the elevated plus maze compared to males [32, 33]. The elevated plus maze is a test of anxiety-like behavior for mice and is analogous to the elevated zero maze. Interestingly, radiation induced more exploration of the open quadrants (suggesting abnormally low anxiety levels and/or increased risky behavior) in the female mice of our study. Pomegranate treatment blocked this effect of radiation on female behavior. Both radiation and pomegranate treatment increased hiding in the enclosed quadrants for male mice, and pomegranate treatment seemed to induce a “female-like” behavioral response in males.

One study reported that exercise-induced elevated levels of hippocampal neurogenesis were correlated with increased anxiety-like behavior on the O-maze (analogous to the zero maze), the open field, and dark/light box, but that radiation attenuated this effect, leading to reduced anxiety-like behaviors in mice, thus suggesting a direct connection between hippocampal neurogenesis and anxiety [34]. It is possible that the antioxidant and anti-inflammatory effects of pomegranate may have ameliorated deleterious effects of radiation on hippocampal neurogenesis in our female mice. Dietary polyphenols have actually been shown to stimulate neurogenesis in the hippocampus [35, 36]. Interestingly, polyphenols found in green tea dose-dependently produced an anxiolytic effect on mice, reducing the amount of time spent in the enclosed areas of the elevated-plus maze [37].

Our findings also demonstrated that pomegranate juice treatment led to gender-specific behavioral changes even in the absence of radiation-induced deficits. Although radiation had no detectable effects on sensorimotor coordination and balance, females performed significantly better than males on the rotarod task [33]. Pomegranate treatment improved the rotarod performance of males to that of females. Again, even though radiation had no effect on the open field test, which provides an assessment of overall activity levels and patterns, pomegranate treatment increased the total distance traveled for male mice, but not female mice, suggesting an effect on exploratory behaviors. Interestingly, most of the gender-specific effects of pomegranate treatment functioned to make the performance of males more similar to the performance of females. Such an effect might be explained by the presence of phytoestrogens in pomegranates [38, 39].

We did not detect a significant effect of radiation or pomegranate treatment on spatial learning and memory as measured by the water maze, but this is consistent with the literature on spatial learning and proton irradiation. One study measured male rats irradiated with a proton beam at 0, 1.5, 3, and 4 Gy and did not see any behavioral differences in spatial learning and memory at any dose [40]. This is in contrast to research on heavy atomic nuclei and high energy level particle (HZE) radiation. Even low doses of HZE, such as 56-Fe, have been shown to cause spatial learning deficits in the water maze [41]. Furthermore, these deficits have been ameliorated by antioxidants found in fruits such as strawberries and blueberries [41], suggesting that HZE particle radiation may cause more extensive hippocampal damage via oxidative stress than proton radiation and that antioxidants may be protective against radiation-induced hippocampal damage. 

## 5. Conclusion

**Figure 5:**
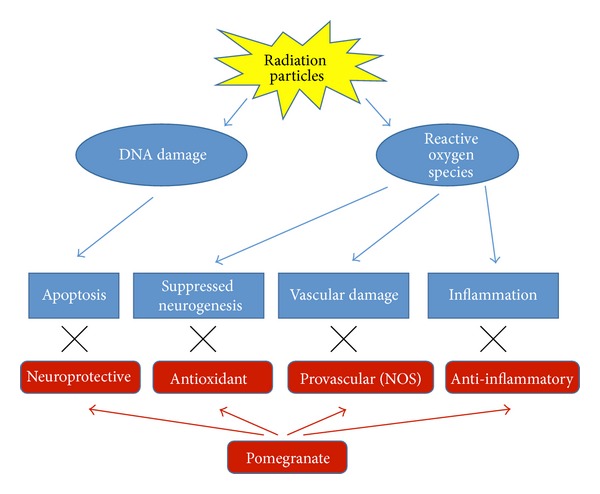
Pomegranates may protect the brain (and therefore behavior) from the effects of radiation by a number of potential mechanisms. Particles can strike DNA, causing damage and apoptosis. Particles can also strike water molecules, generating reactive oxidative species that cause inflammation, vascular damage, and suppressed neurogenesis. Pomegranate's neuroprotective, antioxidant, anti-inflammatory, and provascular (via nitric oxide synthase) properties may protect against these effects.

Our findings suggest that pomegranate therapy may provide protection against radiation-induced changes in affective behaviors, and some of these effects may be gender specific. Furthermore, pomegranates may provide gender specific benefits to sensorimotor coordination. Our findings thus highlight the importance of understanding gender differences in the effects of radiation as well as pomegranate treatment. Women have been part of the space program since its inception and comprise ~11% of the astronauts who have flown in space. Previous research has demonstrated that radiation affects males and females somewhat differently; specific health issues for space travel highlight gender differences in bone loss, orthostatic response, and disease risk, indicating differential effects of the space environment on men and women [42–44]. Altogether, pomegranate consumption seems to induce beneficial effects, and the possible underlying mechanisms should be explored to better understand their putative benefits (Figure 5).
